# Protein aggregation, structural disorder and RNA-binding ability: a new approach for physico-chemical and gene ontology classification of multiple datasets

**DOI:** 10.1186/s12864-015-2280-z

**Published:** 2015-12-16

**Authors:** Petr Klus, Riccardo Delli Ponti, Carmen Maria Livi, Gian Gaetano Tartaglia

**Affiliations:** Gene Function and Evolution, Centre for Genomic Regulation (CRG), Dr. Aiguader 88, 08003 Barcelona, Spain; Universitat Pompeu Fabra (UPF), 08003 Barcelona, Spain; Institució Catalana de Recerca i Estudis Avançats (ICREA), 23 Passeig Lluís Companys, 08010 Barcelona, Spain

**Keywords:** Protein classification, Physico-chemical properties, Gene ontology, Solubility, RNA-binding ability

## Abstract

**Background:**

Comparison between multiple protein datasets requires the choice of an appropriate reference system and a number of variables to describe their differences. Here we introduce an innovative approach to discriminate multiple protein datasets (*multi*CM) and to measure enrichments in gene ontology terms (*clever*GO) using semantic similarities.

**Results:**

We illustrate the powerfulness of our approach by investigating the links between RNA-binding ability and other protein features, such as structural disorder and aggregation, in *S. cerevisiae*, *C. elegans, M. musculus* and *H. sapiens*. Our results are in striking agreement with available experimental evidence and unravel features that are key to understand the mechanisms regulating cellular homeostasis.

**Conclusions:**

In an intuitive way, multiCM and cleverGO provide accurate classifications of physico-chemical features and annotations of biological processes, molecular functions and cellular components, which is extremely useful for the discovery and characterization of new trends in protein datasets. The *multi*CM and *clever*GO can be freely accessed on the Web at http://www.tartaglialab.com/cs_multi/submission and http://www.tartaglialab.com/GO_analyser/universal. Each of the pages contains links to the corresponding documentation and tutorial.

**Electronic supplementary material:**

The online version of this article (doi:10.1186/s12864-015-2280-z) contains supplementary material, which is available to authorized users.

## Background

There is a growing gap between amount of proteomic data and availability of tools for their analysis [1]. While several application programming interfaces are available to analyse computational and experimental results [2], a simple and intuitive interface is currently lacking or missing. Our goal is to start bridging this gap by providing algorithms for analysis of protein sets and discovery of mechanisms that regulate protein function and interactions.

The first method presented here, the *multi*CleverMachine (*multi*CM), is an extension of the *clever*Machine approach (CM [3]) to classify multiple protein datasets using physico-chemical properties. The second algorithm, the *clever*GO, is inspired by the need to simplify Gene Ontology (GO) annotation output. While GO statistics are important to characterize the functional role of proteins, their interpretation is difficult without further downstream processing [2, 4]. Current tools do not provide a unique interface that combines GO term analysis with intuitive interpretation and visualization. For instance, GOrilla [5] calculates GO terms enrichments, but other tools are needed to summarize the results (e.g. REVIGO [6]). *clever*GO integrates multiple analyses in one platform and facilitates GO processing through an interactive analysis accessible via web browser.

We demonstrate the usefulness of our methods by investigating the RNA-binding abilities of *S. cerevisiae* chaperones and their substrates, the physico-chemical determinants of protein insolubility in *S. cerevisiae*, *M. musculus* and *H. sapiens*, and the relationship between aggregation and longevity in *C. elegans*. The purpose of our analysis is twofold: to provide examples that can be used as a reference in other studies and to shed light on the link between nucleic-acid binding abilities and protein features, such as structural disorder and aggregation, that are increasingly recognized as key factors for cellular function and homeostasis [7–9].

## Implementation

The *multi*CM accepts multiple protein sets in FASTA format. Individual sets are classified as positive or negative for binary comparison (the assignment is only needed to create two groups and does not influence the calculations). In each list, the CM screens physico-chemical properties encoded by protein sequences [3] to identify those that best discriminate positive and negative classes (currently supported physico-chemical properties are: nucleic acid binding propensity, membrane propensity, alpha-helix propensity, aggregation propensity, beta sheet propensity, burial propensity and hydrophobicity, but custom properties can be included, as explained in the online Tutorial). For a detailed description of CM performances, we refer to our previous publication [3].

**Fig. 1:**
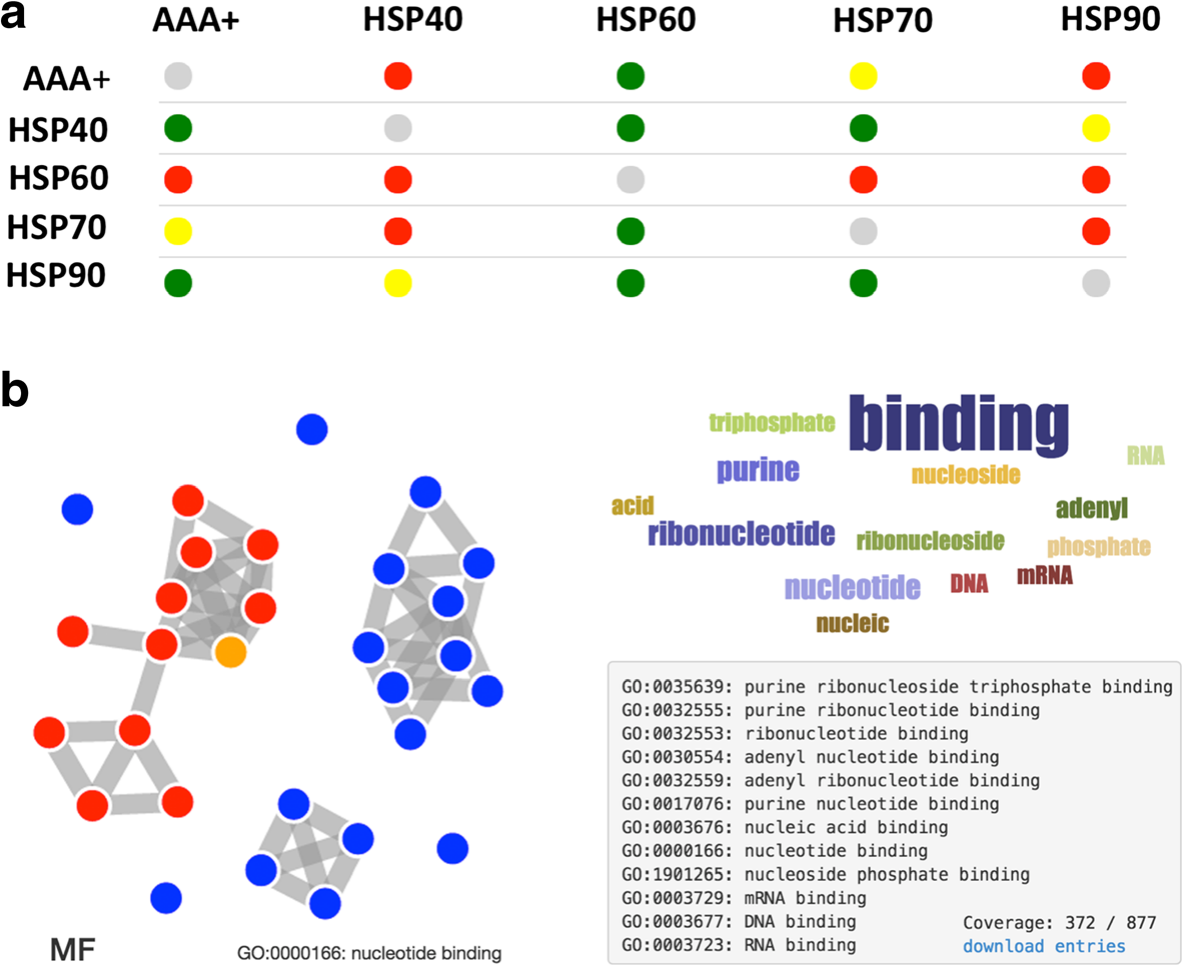
*RNA-binding abilities of S. cerevisiae chaperone substrates.* **a** RNA-binding ability of yeast chaperones substrates is visualized in a microarray-like table. Hsp90 and Hsp40 are predicted to have the largest number of nucleic-acid binding partners (Positive set: vertical axis; Negative set: horizontal axis; Green: positive set is enriched with respect to negative set; Red: negative set is enriched with respect to positive set [3]; Yellow: non significant enrichment; Grey: not calculable enrichment due strong overlap between the sets). The enrichment is associated with a *p*-value < 10^−5^ calculated with Fisher’s exact test. **b** GO annotations are shown through an innovative interface that allows clustering through semantic similarity. The largest cluster of Hsp90 interactors is related to the molecular function (MF) RNA/DNA binding (red cluster corresponding to a coverage of 372 out of 877 proteins). Full analysis is available at http://www.tartaglialab.com/cs_multi/confirm/286/d67c93dd10/

In each *multi*CM run, the information is compiled together from individual models into a high-level overview:

- The user can glean what trend is detected in the data using different physico-chemical features. The indicators collate 10 predictors for each selected feature and represent their consensus with a colour, akin to a *micro-array* slide (Fig. 1a). The colour of each array-spot represents differential states of enrichment for the dataset pairs and allows easy interpretation of increase, decrease or insufficient signal.

The analysis is not restricted to the consensus information only - a link to a full CM view is provided in the main panel (with details on p-value, cross-validation performances, ROC curves and other statistics). The detail view contains ID number of the CM run providing the ability to use it in creation of a *clever*Classifier to study new datasets [3], as well as a link to perform Gene Ontology analysis using the second part of our toolkit, the *cleverGO*.

The *clever*GO webserver provides two ways to explore data:

- The first view of the *clever*GO tool is a classic enrichment table. Enriched GO terms are showed along with coverage, significance and additional information such as the term depth taken from the acyclic GO graph [4]. The enrichment employs interactive filters - users can match text in the description field, sort by significance or exclude terms based on their term depth or precision [10]. Each GO term is linked to AmiGO [11].

In the second *clever*GO visualisation, a force-layout is used to dynamically organize the graph depending on the strength of the connections and separate analyses are generated for biological process, molecular function and cellular component ontologies (Fig. 1b). To illustrate relationships between GO terms and to perform functional clustering, we use semantic similarity [12]. The user can interact with the graph: hover over each node with the cursor yields information about the node, clicking it activates an information panel about the cluster the node belongs to (Fig. 1b). For each of the clusters, *clever*GO shows a list of GO terms that can be individually interrogated, as well as the description of the cluster content. We also provide cluster coverage, i.e. how many of the entries in the user’s submission are annotated with GO terms found in the cluster (the list of entries is also available for the user to download). Each of the operations above is based on the current state of the graph - if the signal strength threshold is changed, the graph’s connectedness changes. If the user applies the minimal term level or precision cut-off, nodes from the graph are filtered. The same principle applies for the *p*-value cut-off (Bonferroni test). Making the graph behaviour dynamic significantly reduces the time needed to perform analysis - the user does not need to re-run any calculation to see the result of a parameter change.

Additional features:

- Upon activation of the detail view on the *multi*CM output page, the user can access the *Boxplotter*. The *Boxplotter* takes the input datasets with best-performing features (passed automatically from the detail view) and shows the distribution of associated propensity scores. On top of the physico-chemical scale information, the *Boxplotter* matches protein IDs with protein abundance databases [13] to provide information on the distribution of expression values. In addition, the *Boxplotter* performs discrimination analysis with the data, showing *p*-values for the statistics and Receiver Operating Characteristic (ROC) curves.

## Results and discussion

To illustrate the performances of both *multi*CM and *clever*GO, we studied the RNA-binding abilities of *S. cerevisiae* chaperone substrates [14], the physico-chemical determinants of protein insolubility in in *S. cerevisiae*, *M. musculus* and *H. sapiens* [15], and the link between protein aggregation and longevity in *C. elegans* [16].

### RNA-binding abilities of *S. cerevisiae* chaperone substrates

Systematic analysis of physical TAP-tag based protein-protein interactions revealed individual networks of *S. cerevisiae* chaperones [14]. In agreement with experimental evidence, the *multi*CM predicts that Hsp90 (Hsp82) [17] and Hsp40 (Cwc23) [18] are prone to associate with RNA-binding proteins (RBPs; Fig. 1a; red dots indicate enrichment over other chaperones). By contrast, Hsp60 shows the lowest propensity to interact with RBPs, which is consistent with its main role of guiding hydrophobic proteins to fold into the native state [19] (Fig. 1a; green dots indicate depletion over other chaperones). Moreover, Hsp70 (Ssb1) binds directly with transcripts and is predicted to have more RBP partners than Hsp60 [20]. AAA+ (Hsp78) shows similar pattern as Hsp70, in agreement with the fact that the two chaperones work together [21]. As for other physico-chemical features, *multi*CM reports that both Hsp40 and Hsp78 associate with structurally disordered (and hydrophilic [22]) proteins, which is in line with previous experimental studies on prion propagation [23], while Hsp60, Hsp70 and Hsp90 are predicted to bind to hydrophobic proteins [3, 19]. To further investigate Hsp90 features, we performed *clever*GO analysis of its substrates. Looking at the molecular function (Fig. 1b), we observe an enrichment in GO terms related to RBPs (e.g., class “RNA-binding” shows *p*-value < 10^−5^; Bonferroni test), which very well complements our predictions of physico-chemical features. Importantly, the nucleic-acid cluster is the largest in terms of dataset coverage (>40% of the substrates list; Fig. 1b).

### Physico-chemical determinants of protein insolubility

**Fig. 2:**
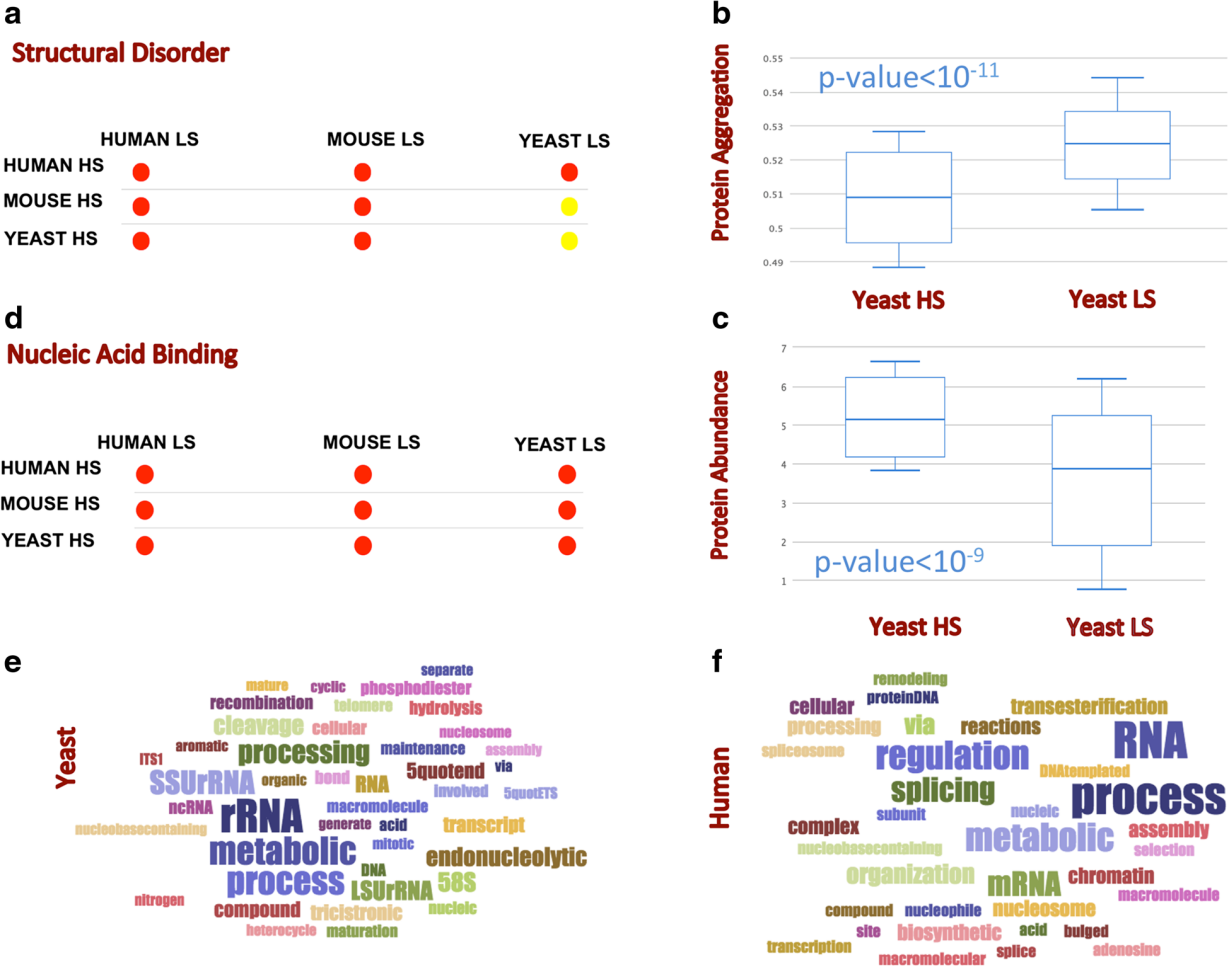
*Physico-chemical determinants of protein insolubility.* Comparing low-solubility (LS) and high-solubility (HS) proteins in three eukaryotic cells [15], we found that **a** LS proteins are structurally disordered in human and mouse (red dots indicate enrichments in LS proteins).**b** The *Boxplotter* algorithm indicates that there is a significant difference between aggregation-propensities of HS and LS groups in yeast (*p*-value = 10^−11^; Mann–Whitney–Wilcoxon test; area under the ROC curve = 0.72), which is **c** inversely related to protein abundance (*p*-value = 10^−9^; Mann–Whitney–Wilcoxon test; area under the ROC curve = 0.70), in agreement with previous evolutionary observations [30–32]. In all organisms, we find **d** more nucleic acid binding in LS fractions. **e**, **f** LS proteins are enriched in nucleic-acid binding ability (Additional file 1: Figure S1), as shown with *clever*GO analysis on human and yeast. The links to *multi*CM, *Boxplotter* and *clever*GO analyses are available at http://www.tartaglialab.com/cs_multi/confirm/737/6065feed14/

A recent mass-spectrometry study investigated protein precipitates formed upon centrifugation of *S. cerevisiae*, *M. musculus* and *H. sapiens* cells [15]. Two major determinants have been reported to promote insolubility: structural disorder in *H. sapiens* and *M. musculus*, which induces aberrant interactions promoting precipitation of protein complexes [24], and aggregation propensity [25] in *S. cerevisiae* cells, which is linked to the presence of hydrophobic residues exposed on protein surfaces [22]. Using the *multi*CM approach to compare low-solubility (LS) and high-solubility (HS) proteins, we observed that *H. sapiens* and *M. musculus* have a larger fraction of structurally disordered regions in the LS group, while non-significant enrichments were found in yeast (Fig. 2a). Differently from *H. sapiens* and *M. musculus* cells*, S. cerevisiae* shows high intrinsic aggregation propensity (i.e., calculated in the unfolded state) for LS proteins (Fig. 2b), in agreement with analyses carried out with TANGO [26] and AGGRESCAN [27] performed in the original study [15]. Yet, the HS group has higher burial in *H. sapiens* and *M. musculus* (Additional file 1: Figure S1A), which suggests that aggregation-prone amino acids are less abundant on surfaces when proteins are natively folded [28, 29]. In addition to discriminating LS and HS groups in *S. cerevisiae* (*p*-value = 10^−11^; Mann–Whitney–Wilcoxon test; Area under the ROC curve = 0.72; Fig. 2b) the aggregation propensity is also anti-proportional to protein abundance (*p*-value = 10^−9^; Mann–Whitney–Wilcoxon test; Area under the ROC curve = 0.70; Fig. 2c), which is in line with previous observations suggesting an evolutionary pressure to reduce the expression of amyloidogenic proteins [30–32]. In agreement with GO analysis performed in the experimental study [15], we found strong enrichment of RBPs in the LS proteins of human (e.g., class “RNA-binding” has *p*-value < 10^−8^; Bonferroni test), mouse (“RNA-binding” with *p*-value < 10^−8^) and yeast (“RNA-binding” with *p*-value < 9*10^−8^) cells*,* supporting  the hypothesis that RNA molecules provide the scaffold for protein interactions [33] and (Fig. 2d, e and f).

### Protein aggregation and longevity

**Fig. 3:**
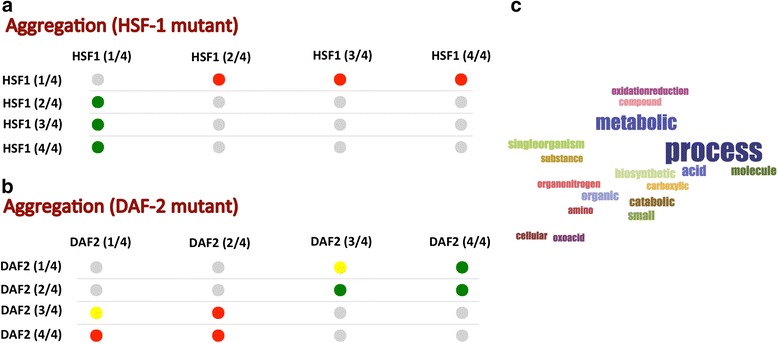
*Protein aggregation and longevity.* We used *multi*CM to analyze insoluble fractions of *C. elegans* proteins [16]. **a** Analysis of mass-spectrometry data indicates that in the *hsf-1* strain (short-lived) highly enriched proteins (class HSF 4/4) are more aggregation prone than those less enriched (class HSF1 1/4). **b** In the *daf-2* strain (long-lived), highly enriched proteins (DAF2 4/4) show lower aggregation propensities than the ones poorly enriched (DAF2 1/4). In these calculations, the insoluble fraction of the strains is divided into 4 equal sets containing proteins with fold enrichments > 1 with respect to wild type worm and ranked from low (1/4) to high (4/4)  [green dots indicate row vs column enrichments]. **c** Using the *clever*GO algorithm, we analyzed proteins present in the *hsf-1* strain (i.e., reported in HSF-1 4/4 and not in DAF-2 4/4) and found enrichments in metabolic pathways, oxidative stress response and mitochondrial function. Links to the analyses are at http://www.tartaglialab.com/cs_multi/confirm/757/9e1710f579/ and http://www.tartaglialab.com/cs_multi/confirm/758/95acfc44da/

It has been observed that inhibition of the insulin growth 1 signaling pathways leads to a dramatic lifespan extension of *C. elegans* strains carrying mutation in the *daf-2* receptor and that transcription factor *hsf-1* is essential for longevity [16]. Mass-spectrometry analysis of long-lived *daf-2* and short-lived *hsf-1* mutant strains revealed two major types of deposits that accumulate during aging: *hsf-1* mutant proteins have high aggregation propensities, while *daf-2* mutant proteins show decreased structural content [16]. Thus, decrease in longevity can be associated with accumulation of aggregation-prone proteins, whereas lower hydrophobicity is linked to different type of deposits and significantly reduced toxicity. Using the *multiCM* approach to compare the insoluble fraction of *hsf-1* mutant strain with wild type worm (WT), we found that proteins showing high enrichment in mass-spectrometry analysis (class HSF-1 4/4) are more aggregation-prone than those with low enrichment (class HSF-1 1/4) [Fig. 3a]. By contrast, proteins enriched in *daf-2* mutant worms (DAF-2 4/4) have lower aggregation propensities than those showing low enrichment (DAF-2 1/4). In the *daf-2* mutant strain (DAF-2 3/4 and DAF-2 4/4) enrichments are associated with decrease in beta-sheet content (Additional file 1: Figure S2A), while in *hsf-1* mutant worms (HSF-1 3/4 and HSF-1 4/4) we observe depletion of structural disorder (Additional file 1: Figure S2B). Proteins present in the *hsf-1* strain (i.e., listed in HSF-1 4/4 and not included in DAF-2 4/4) are involved in several metabolic processes (e.g., class “oxidative stress response” with *p*-value < 6*10^−4^; Bonferroni test), oxidative stress response (e.g., class “metabolic process” shows *p*-value < 10^−7^) and mitochondrial function (e.g., class “mitochondrion” with *p*-value < 10^−7^), as reported in the original study (Fig. 3c) [16]. In addition, and in line with the work on *S. cerevisiae*, *M. musculus* and *H. sapiens* proteomes [15], we found an enrichment of RBPs (e.g., class “RNA-binding” shows *p*-value = 7*10^−3^), which reinforces the link between protein deposition and nucleic acid binding [34].

## Conclusions

In this work, we introduced two innovative approaches to compare multiple protein datasets using physico-chemical properties and GO annotations: the *multi*CM allows feature classification and the clever*GO* provides clustering through semantic relationships. We illustrated the performances of both *multi*CM and clever*GO* using examples related to RNA-binding abilities of *S. cerevisiae* chaperone substrates [14], physico-chemical determinants of protein insolubility in *S. cerevisiae*, *M. musculus* and *H. sapiens* [15] and the link between aggregation and life-span in *C. elegans* [16]. In all cases, the results are in agreement with available evidence on protein functions and interactions, providing a clear indication on the flexibility and broad applicability of our algorithms.

As shown in the examples, we are particularly interested in understanding the relationship between nucleic-acid binding ability and structural disorder and aggregation. Indeed, previous studies indicate that RNA secondary structures [35], especially when enriched in GC content [36], contribute to spatial rearrangement of disordered regions, promoting the formation of protein-RNA complexes. In agreement with these observations, it has been reported that intrinsically disordered proteins interact with RNA [8, 37], which influences protein aggregation [38] and, in turn, toxicity [39]. The involvement of nucleic acid molecules in protein aggregation [40] is compatible with the findings discussed in our examples and provides an intriguing working hypothesis [7, 41] to study neurodegenerative events [42] that are characterized by aggregation [43] and structural disorder [44]. As a matter of fact, previous work indicates that presence of polyanions lead to reduction of protein stability [45] and nucleic acids have a strong tendency to accumulate in neurofibrillary tangles and senile plaques [46]. Recent evidence also shows that aggregation-related mutations in the RBPs Tar DNA-binding protein 43 TDP-43 and Translocated in liposarcoma protein FUS are associated with the formation of RNA granules [47, 48] that are phase separated, non-membrane-bound ribonucleoprotein aggregates [49, 50].

In conclusion, theoretical approaches for prediction of protein features, such as those integrated in the *multi*CM for prediction of structural disorder, aggregation and nucleic-acid binding ability [51–53], will be useful to provide insights into functional networks. We hope that our tools will be useful for the discovery of trends in protein datasets, complementing experimental [54, 55] and theoretical analyses [31, 56–58].

## Availability and requirements

The *multi*CM and *clever*GO are available at http://www.tartaglialab.com/cs_multi/submission and http://www.tartaglialab.com/GO_analyser/universal.

Tutorials can be accessed at http://www.tartaglialab.com/cs_multi/tutorial and http://www.tartaglialab.com/GO_analyser/tutorial. Documentation files are deposited at http://service.tartaglialab.com/static_files/algorithms/clever_machine/documentation.html.

## Additional file

Additional file 1: Figure S1.
*Physico-chemical determinants of protein insolubility.* High-solubility (HS) proteins show A) higher burial in human and mouse, in agreement with the observations reported in the original study. **Figure S2.** *Physico-chemical of C. elegans mutant strains.* A) In the *hsf-1* strain, highly enriched proteins (HSF 4/4) are less structurally disordered than those poorly enriched (HSF1 1/4). B) In the *daf-2* strain (long-lived), highly enriched proteins (DAF2 4/4) show lower beta-sheet propensities than those poorly enriched (DAF2 1/4), in agreement with observations reported in the original experimental study. (DOCX 412 kb)
