# Supplementary material for: Protein aggregation, structural disorder and RNA-binding ability: a new approach for physico-chemical and gene ontology classification of multiple datasets
Source: BMC Genomics. 2015 Dec 16;16:1071. doi: 10.1186/s12864-015-2280-z (PMC4681139; doi:10.1186/s12864-015-2280-z)
Supplement: Additional file 1: Figure S1. — Physico-chemical determinants of protein insolubility. High-solubility (HS) proteins show A) higher burial in human and mouse, in agreement with the observations reported in the original study. Figure S2. Physico-chemical of C. elegans mutant strains. A) In the hsf-1 strain, highly enriched proteins (HSF 4/4) are less structurally disordered than those poorly enriched (HSF1 1/4). B) In the daf-2 strain (long-lived), highly enriched proteins (DAF2 4/4) show lower beta-sheet propensities than those poorly enriched (DAF2 1/4), in agreement with observations reported in the original experimental study. (DOCX 412 kb) [file 12864_2015_2280_MOESM1_ESM.docx]

**Supplementary Material**

**
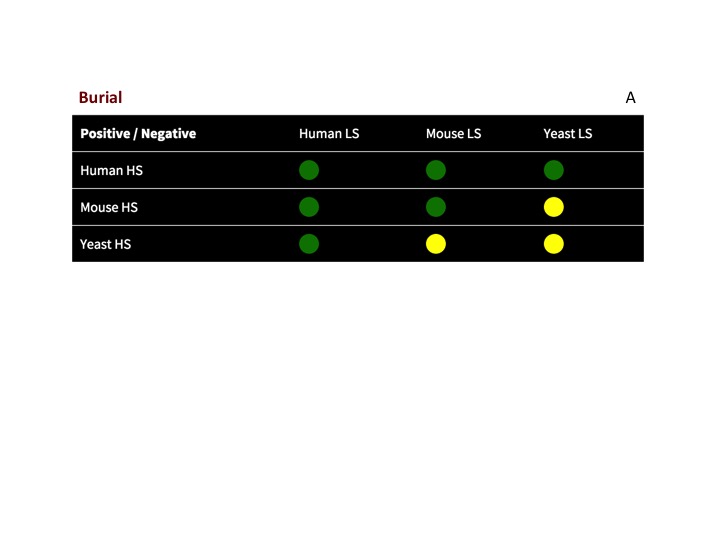
**

**Supplementary Figure 1.** *Physico-chemical determinants of protein insolubility.* High-solubility (HS) proteins show A) higher burial in human and mouse, in agreement with the observations reported in the original study.

**Supplementary Figure 2.** *Physico-chemical of C. elegans mutant strains.* A) In the *hsf-1* strain, highly enriched proteins (HSF 4/4) are less structurally disordered than those poorly enriched (HSF1 1/4). B) In the *daf-2* strain (long-lived), highly enriched proteins (DAF2 4/4) show lower beta-sheet propensities than those poorly enriched (DAF2 1/4), in agreement with observations reported in the original experimental study.
